# The Use of Information Entropy and Expert Opinion in Maximizing the Discriminating Power of Composite Indicators

**DOI:** 10.3390/e26020143

**Published:** 2024-02-06

**Authors:** Matheus Pereira Libório, Roxani Karagiannis, Alexandre Magno Alvez Diniz, Petr Iakovlevitch Ekel, Douglas Alexandre Gomes Vieira, Laura Cozzi Ribeiro

**Affiliations:** 1 Graduate Program in Computer Science, Pontifical Catholic University of Minas Gerais, Belo Horizonte 30535-901, Brazil; petr.ekel2709@gmail.com (P.I.E.); laura.cozzi.ribeiro@gmail.com (L.C.R.); 2 Center for Planning and Economic Research, 11 Amerikis Str., 10672 Athens, Greece; rkarag@kepe.gr; 3 Graduate Program in Geography, Pontifical Catholic University of Minas Gerais, Belo Horizonte 30535-901, Brazil; alexandremadiniz@gmail.com; 4 Graduate Program in Mathematical Modeling, Federal Center of Technological Education of Minas Gerais, Belo Horizonte 30421-169, Brazil; douglas.agv@gmail.com

**Keywords:** composite indicators, information entropy, cost of doing business, discriminating power, hybrid weighting scheme

## Abstract

This research offers a solution to a highly recognized and controversial problem within the composite indicator literature: sub-indicators weighting. The research proposes a novel hybrid weighting method that maximizes the discriminating power of the composite indicator with objectively defined weights. It considers the experts’ uncertainty concerning the conceptual importance of sub-indicators in the multidimensional phenomenon, setting maximum and minimum weights (constraints) in the optimization function. The hybrid weighting scheme, known as the SAW-Max-Entropy method, avoids attributing weights that are incompatible with the multidimensional phenomenon’s theoretical framework. At the same time, it reduces the influence of assessment errors and judgment biases on composite indicator scores. The research results show that the SAW-Max-Entropy weighting scheme achieves greater discriminating power than weighting schemes based on the Entropy Index, Expert Opinion, and Equal Weights. The SAW-Max-Entropy method has high application potential due to the increasing use of composite indicators across diverse areas of knowledge. Additionally, the method represents a robust response to the challenge of constructing composite indicators with superior discriminating power.

## 1. Introduction

Composite indicators are aggregations of normalized sub-indicators, whether weighted or not designed to facilitate understanding complex multidimensional realities. They aid managers, researchers, and academics in decision making [1]. The literature on composite indicators is voluminous and diverse, addressing theoretical and methodological issues in depth [2,3].

The range of applications of composite indicators is broad, offering solutions to multidimensional nature problems in diverse knowledge fields. These include sustainability [4], community resilience [5], environment [6], healthcare [7], quality of work [8], tourism [9], transport [10], education [11], spatial inequality [12], pandemic impact assessment [13], and vulnerability to food consumption [14], among many others [15,16].

Much of the specialized literature is focused on improving traditional methods for constructing composite indicators [17,18]. In general, traditional statistical and multi-criteria methods for constructing composite indicators fail in some way, especially in the sub-indicators weighting and aggregation process [1,19,20,21].

The endogenous sub-indicator weighting scheme using statistical methods such as Principal Component Analysis and Benefit-of-the-Doubt does not guarantee compatibility with the theoretical framework of multidimensional phenomenon [22,23]. In contrast, the exogenous sub-indicators weighting scheme using multi-criteria methods such as the Budget Allocation Process, Analytic Hierarchy Process, and Ordered Weighting Average faces widespread criticism due to its susceptibility to assessment errors and judgment biases [24,25]. Furthermore, statistical and multi-criteria methods that employ the sub-indicators compensatory aggregation approach for sub-indicators generate undesirable results and allow compensation (substitutability) between poor and above-average performance sub-indicators [26].

Although current advances do not completely solve the lack of a weighting scheme and a perfect aggregation approach [26], these advances address important issues. They offer more realistic representations of multidimensional phenomena by reducing information loss during the aggregation of sub-indicators [27,28], considering spatial autocorrelation [29]; concerning discrepant or out-of-scale data [30], avoiding compensation between poor and above-average performance sub-indicators [31], and increasing the composite indicator discriminating power [32], among others [33]. This research focuses on improving methods that construct composite indicators based on Shannon’s [34] information theory.

On the one hand, Shannon’s [34] information Entropy Index offers a valuable measure of informational diversity [35,36] with high applicability in the endogenous sub-indicator weights definition [37,38] and in reducing informational loss arising from the sub-indicators aggregation [28,39]. Other examples of informational diversity measures are the Gini and the variation coefficients [40].

Beyond serving as a weighting scheme free from assessment errors and judgment biases [41], the information Entropy Index has a high degree of usefulness in increasing the discriminating power of composite indicators constructed using frontier-based methods such as Data Envelopment Analysis and Benefit-of-the-Doubt [42,43].

Conversely, composite indicators constructed based on information entropy exhibit shortcomings in at least three ways. First, endogenous weighting schemes disregard the conceptual importance of sub-indicators in the multidimensional phenomenon, resulting in weights incompatible with the multidimensional phenomenon theoretical framework [22,23]. Second, sub-indicators aggregation prevents attributing greater weights to sub-indicators with greater informational diversity, thereby failing to ensure sufficient informational power for the composite indicator [26]. Third, the fully compensatory aggregation (arithmetic mean) of the sub-indicators modifies the function of the weights, rendering them no longer a measure (coefficient) of importance [20].

This research aims to develop a method that combines expert opinion with the discriminating power (Entropy Index) of the composite indicator in the endogenous definition of weights. The method uses the Simple Additive Weighting (SAW) framework and can be summarized as the harmonic aggregation of normalized sub-indicators weighted with weights that maximize the composite indicator entropy.

The method so-called SAW-Max-Entropy addresses a gap by providing solutions capable of enhancing the discriminating power of composite indicators while simultaneously assigning weights to sub-indicators free from errors and judgment biases yet compatible with their relative importance in the multidimensional phenomenon concept. This innovative solution makes SAW-Max-Entropy a pioneering method in constructing composite indicators, achieving a more significant distinction between decision-making units and improving the ability of the composite indicator to portray multidimensional phenomena such as well-being [42], efficiencies of hotel chains [43], and water quality evaluation [38].

The primary contribution of the research is to highlight that sub-indicator aggregation undermines the objective of increasing the discriminatory power of the composite indicator by attributing greater weights to sub-indicators with higher informational diversity. This is an impactful finding, considering the widespread use of the Entropy Index weighting scheme to increase the discriminating power of composite indicators (e.g., [41,44,45,46,47]. This evidence makes SAW-Max-Entropy an advantageous method for increasing the discriminating power of composite indicators, as demonstrated by the example of the Cost of Doing Business Index [48].

The remaining parts of this research are organized as follows. Section 2 describes the SAW-Max-Entropy method. Section 3 presents the Cost of Doing Business Index, the data collection procedures for the sub-indicators (Section 3.1), the definition of the sub-indicator weights by expert, maximum, minimum, group, and consensus degree (Section 3.2), the metrics for analyzing results (Section 3.3) and an analysis and discussion of results (Section 3.4). The research conclusions, including limitations and lines of future investigation, are presented in Section 4.

## 2. SAW-Max-Entropy Method

The motivation behind developing SAW-Max-Entropy stems from the observation that subjective weighting of sub-indicators by expert opinion can lead to the construction of more stable composite indicators in certain situations compared to objective weighting through methods such as the Entropy Index and Principal Component Analysis (see [44]).

The design of SAW-Max-Entropy is inspired by Karagiannis and Karagiannis [37], who propose a weighting scheme whereby greater weights are assigned to sub-indicators with higher informational diversity (variation), thereby increasing the discriminating power of the composite indicator and facilitating the decision process. Like Karagiannis and Karagiannis [37], SAW-Max-Entropy is designed to maximize discriminating power but with a specific focus on the composite indicator scores, aiming to enhance the distinction between decision-making units and improve the decision process.

The core of SAW-Max-Entropy lies in its hybrid weighting scheme, which considers subjective information while objectively defining the weights. Hybrid weighting schemes, wherein expert opinion is considered in the objective definition of weights, are frequently used in several studies [49,50,51,52].

The fundamental difference between SAW-Max-Entropy and the previously presented methods lies in the objective of maximization. While other methods aim to maximize scores of the composite indicator [28], the variance extracted from the sub-indicators [49], the composite indicator external validity [50], and the composite indicator spatial autocorrelation [53], SAW-Max-Entropy aims to maximize informational diversity (Entropy Index), specifically focusing on enhancing the discriminating power of the composite indicator.

The operationalization of SAW-Max-Entropy is quite simple, consisting of the harmonic aggregation of normalized and weighted sub-indicators:

(1) $$\alpha_{\delta }=\left[{\left(\frac{\sum_{\lambda =1}^{\eta }\varpi_{\lambda }\beta_{\lambda }^{-1}}{\sum_{\lambda =1}^{\eta }\varpi_{\lambda }}\right)}^{-1}\right]$$

where $\alpha_{\delta }$ is the composite indicator score of the δ-th decision-making unit; $\beta_{\lambda }$ is the λ-th normalized sub-indicator, $\varpi_{\lambda };\;\lambda =1,\;2,\ldots ,\eta$ is the vector of sub-indicator weights that satisfies the following conditions $\varpi_{\lambda }\in \left[0,1\right];\;\lambda =1,\;2,\ldots ,\eta$ and $\sum_{\lambda =1}^{\eta }\varpi_{\lambda }=1$.

The weights used in (1), which maximize the composite indicator Entropy Index, are obtained from the solution of the following problem:

(2) $$-\frac{1}{\ln\psi }\overset{\psi }{\underset{\delta =1}{\sum }}\alpha_{j}\ln\alpha_{\delta }\to \underset{\varpi_{\lambda }^{-}\leq w_{\lambda }\leq \varpi_{\lambda }^{+}}{\max}$$

where $\varpi_{\lambda }^{-}$ is the lowest weight among the weights $\varpi_{\lambda }\;$defined by the experts, $\varpi_{\lambda }^{+}$ is the highest weight among the weights $\varpi_{\lambda }$ defined by the experts, and ψ is the number of decision-making units.

## 3. Application Example: Costs of Doing Business in G20 Countries

The Cost of Doing Business Index is a composite indicator created by Bernardes et al. [48] to measure the cost structure incident on companies when carrying out their economic activities. In addition to offering a measure of the costs borne by countries’ entrepreneurs, the index is valuable for identifying the costs that most impact companies. It indicates which reforms governments should prioritize to stimulate business activity and enhance economic performance [54].

The Cost of Doing Business Index comprises seventeen cost sub-indicators selected among the forty-one sub-indicators of the World Bank’s [55] Ease of Doing Business Index. Although the Ease of Doing Business Index is more comprehensive and widely recognized, its extensive number of sub-indicators increases cognitive stress for experts when assessing weights, resulting in errors and inconsistencies [20].

At this point, it is necessary to recognize that assigning weights to seventeen sub-indicators poses its own challenges [24]. The natural strategy to overcome this challenge is the application of so-called transformation functions [56,57,58,59]. This strategy enables experts to choose their preferred criteria/alternative assessment format, e.g., ordering, scoring, comparison, or budget allocation [60], giving them greater psychological comfort and reducing errors and inconsistencies in assessments [51].

### 3.1. Sub-Indicator Data Collection

Data for the seventeen cost sub-indicators from the latest EDBI report were extracted from the World Bank Databank (https://databank.worldbank.org/, accessed on 23 January 2024). These include resolving insolvency (Ri1); starting a business (St1 St2); building permits (Cp1); registering property (Rp1); obtaining energy (Ge1); legal fees (Ec1), contract fees (Ec2), court costs (Ec3), and contract execution costs (Ec4); border compliance to export (Tb1), and documentary compliance to export (Tb2); border compliance to import (Tb3) and documentary compliance to import (Tb4); labor fees (Pt1), profits fees (Pt2), and total tax and contribution rate (Pt3).

Then, the data were normalized and used in (1). The maximization normalization function was implemented, as the relationship of the sub-indicators with the Cost of Doing Business Index is positive [61]. This function was adapted to allow harmonic aggregation when the normalization result is equal to zero:

(3) $$\beta_{\lambda }=\frac{\beta_{\lambda \delta }-\min\left(\beta_{\lambda \delta }\right)}{\max\left(\beta_{\lambda \delta }\right)-\min\left(\beta_{\lambda \delta }\right)}+\pi$$

where $\beta_{\lambda \delta }$ is the score of the λ-th sub-indicator of the δ-th decision-making unit, and π is a small constant that guarantees that $\beta_{\lambda }$ is greater than zero.

### 3.2. Sub-Indicator Weights: By Expert, Maximum, Minimum, Group, and Consensus Degree

The sub-indicator weights used in (2) were obtained based on the opinion of ten experts in international business. Experts were instructed to rank the sub-indicators in order of importance to reduce cognitive stress during the assessment process [51].

Then, the rankings were transformed into weights using the Rank Reciprocal function [62,63]. This function has the main advantage of obtaining weights that are more compatible with the opinion of the group of experts [24], which are operationalized as follows:

(4) ϖλ=1λ∑λ=1η1θλ , λ1,2,.., η

where $\theta_{\lambda }$ is the order of importance of the λ-th sub-indicator.

Based on the $\theta_{\lambda }$ defined by each of the experts $\varepsilon^{\rho }$, it is possible to obtain a consensus degree among the group of experts [24]:

(5) $$\phi =\frac{\sum_{\lambda =1}^{\eta }\sum_{\varepsilon =1}^{\rho }1-\left(\frac{\left|\phi_{\left(\theta_{\lambda }\right)}-{\varepsilon^{\rho }}_{\left(\theta_{\lambda }\right)}\right|}{\eta -1}\right)}{\eta }$$

where $\phi_{\left(\theta_{\lambda }\right)}$ is the position of the λ-th sub-indicator according to the group of experts, ${\varepsilon^{\rho }}_{\left(\theta_{\lambda }\right)}$ is the position of the λ-th sub-indicator according to experts $\varepsilon^{\rho }$.

**Figure 1:**
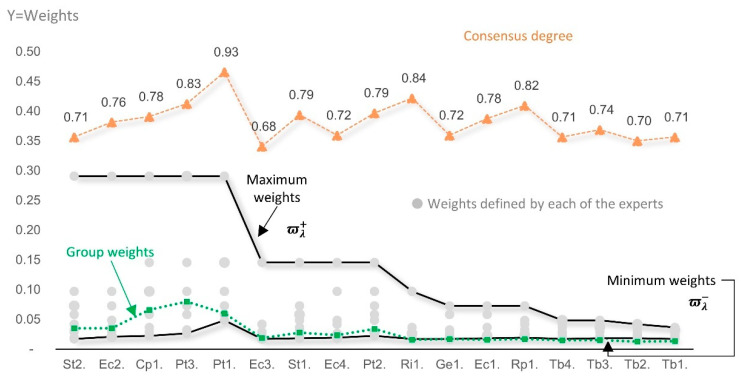
Weights of sub-indicators (by an expert, group of experts, maximum and minimum) and consensus degree on the weights. Note: the consensus degree is not linked to the *Y*-axis scale.

Figure 1 shows the results of the assessment of the sub-indicators by experts, indicating the weights by experts (points), maximum and minimum weights (points on solid lines), weights according to the group of experts (squares on the dotted line), and consensus degree (triangles on the dotted line).

The maximum ($\varpi_{\lambda }^{+}$) and minimum ($\varpi_{\lambda }^{-}$) weights assigned to each sub-indicator, according to the experts’ opinion, reflect the range over which the weights in (2) can vary while maximizing the composite indicator Entropy Index. Therefore, the lower the consensus degree among experts concerning a sub-indicator weight, the greater the uncertainty concerning its relative importance in the composite indicator, and the greater the potential of the SAW-Max-Entropy method in increasing the composite indicator entropy index, that is, its discriminating power.

### 3.3. Metrics for Analyzing Results

Informational diversity (Section 2), linkage with external variables, uncertainty analysis, and consensus degree (Section 3.2) were the four metrics used to analyze and compare the composite indicator constructed by the Max-Entropy weighting scheme with those composite indicators constructed by the Entropy Index, Expert Opinion, and Equal Weights weighting schemes.

The link with external variables and uncertainty analysis are two metrics frequently used to attest to the ability of the composite indicator to capture the multidimensional phenomenon and to verify the stability of its internal structure [1].

Link with external variable: correlation of the composite indicator with a conceptually relevant variable of the multidimensional phenomenon, as is the case with Gross Domestic Product (GDP) per capita and the costs of doing business in the countries [48].

Uncertainty analysis: average deviations in the positions of decision-making units in the composite indicators ranking constructed in different ways, as is the case with composite indicators constructed with sub-indicators weighted using Shannon Entropy, Expert Opinion, Max-Entropy, and Equal Weights.

### 3.4. Analysis and Discussion of Results

**Figure 2:**
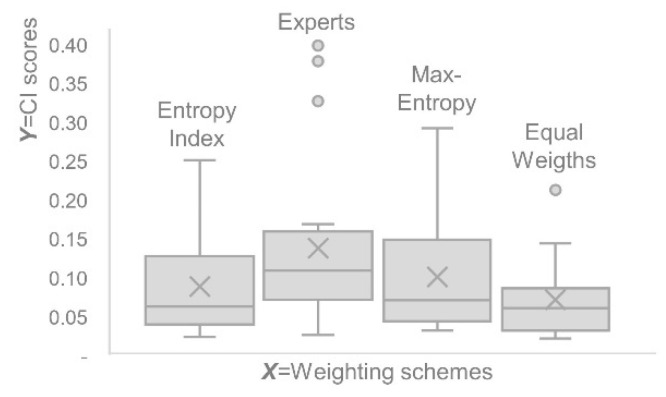
Dispersion of composite indicator scores constructed by the Entropy Index, Expert Opinion, Max-Entropy, and Equal Weights weighting schemes.

First, let us compare the discriminating power of the composite indicators constructed by different weighting schemes. Note in Figure 2 that the composite indicator scores constructed by the Max-Entropy weighting scheme present greater dispersion than those constructed by the Entropy Index, Expert Opinion, and Equal Weights weighting schemes. This increased dispersion is helpful to differentiate decision-making units, aiding the decision-making process. Furthermore, none of the decision-making units of the composite indicator constructed by SAW-Max-Entropy presents scores with atypical values, as observed in composite indicators constructed with sub-indicators weighted using Expert Weights and Equal Weights.

The higher discriminating power of the composite indicator constructed by the SAW-Max-Entropy method is also evident through its informational diversity. Table 1 shows that the informational diversity of the composite indicator constructed by SAW-Max-Entropy is 1.38, 1.15, and 1.24 times greater than that of the composite indicators constructed by the Entropy Index, Expert Opinion, and Equal Weights weighting schemes, respectively.

The SAW-Max-Entropy method also offers a better solution concerning the link with external variables (correlation with GDP) and consensus degree metrics compared to composite indicators constructed by the Entropy Index and Equal Weights weighting schemes.

The results indicate that the objective weighting of sub-indicators by the Entropy Index, like other objective weighting methods, assigns weights that are incompatible with the conceptual importance of the sub-indicators [22,23]. Table 2 shows that 41% of the weights defined by the Entropy Index weighting scheme exceed the maximum weights or fall below the minimum weights defined by the experts. Furthermore, the Entropy Index sub-indicator weighting negatively impacts the discriminating power and the composite indicator’s capacity to capture the multidimensional phenomenon concept, as evidenced by the metrics of informational diversity and correlation with GDP.

The findings of this research reveal that the SAW-Max-Entropy method effectively addressed the challenge of objectively finding weights compatible with the experts’ opinion regarding the conceptual importance of sub-indicators in the multidimensional phenomenon. This hybrid weighting scheme offers a balanced solution across all metrics analyzed. In particular, the SAW-Max-Entropy method proved efficient in maximizing the discriminating power of the compositive indicator without disregarding the expert’s opinion in sub-indicator weighing.

**Figure 3:**
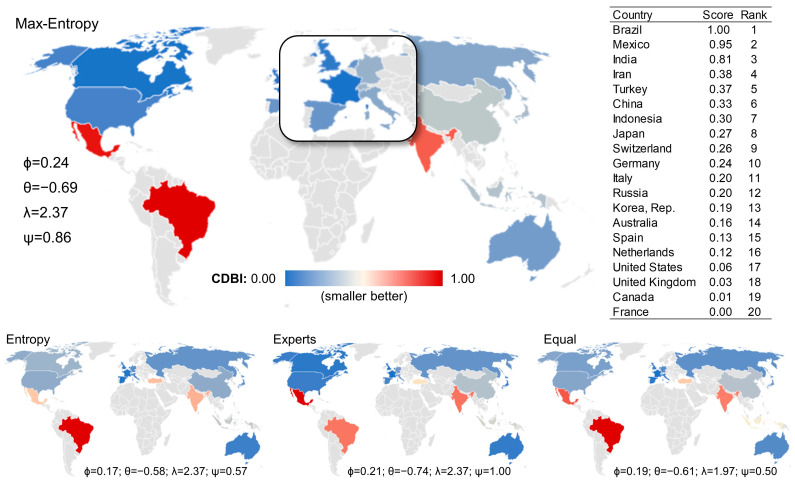
Cost of Doing Business Index for G20 countries. Note: see the complete data on scores and rankings in Table A1 and Table A2 of Appendix A.

In this regard, the SAW-Max-Entropy method plays a crucial role in analyzing business costs in G20 countries (Figure 3), making it easier to distinguish between countries concerning their business costs. It also offers a consistent representation of the multidimensional phenomenon, as evidenced by the negative correlation between business costs and countries’ economic performance. In addition, varying sub-indicator weights do not result in significant shifts in the ranking of the decision-making units, signaling a stable internal structure. Finally, it reliably indicates the sub-indicators that impact the composite indicator scores most, offering more precise information on which reforms governments should prioritize to stimulate economic activity.

The results, including the complete data on scores and rankings in Table A1 and Table A2 of Appendix A, reveal that the SAW-Max-Entropy method can be employed to improve the ability of the Cost of Doing Business Index to portray the regulatory environment of countries, offering a more accurate signal for attracting foreign direct investment [48]. The method also has a high potential to precisely identify the sub-indicators that most impact the Cost of Doing Business Index, helping governments prioritize reforms [54]. Similarly, the SAW-Max-Entropy method can contribute to improving the quantification and monitoring of countries’ ease of doing business [33], identifying areas for opening new companies with greater precision [64], offering more realistic comparisons of countries’ ease of doing business [65], and enhancing understanding of the relationship between the ease of doing business and regulatory barriers and controls on the movement of capital in countries [66], among others (e.g., [67]).

The results of this research also contribute significantly to the literature on composite indicators. In particular, it reveals that the sub-indicator aggregation undermines the expected increase in the discriminating power of the composite indicator by attributing greater weights to sub-indicators with greater informational diversity. This finding is particularly impactful given the widespread adoption of the Entropy Index weighting scheme in numerous studies [41,44,45,46,47,68,69]. This outcome raises the importance of SAW-Max-Entropy as a method capable of increasing the discriminating power of composite indicators and overcoming a limitation of objective weighting methods, namely the attribution of weights incompatible with the conceptual importance of sub-indicators.

These properties make SAW-Max-Entropy a promising method to increase the discriminating power of composite indicators in various domains, including well-being [42], efficiencies of hotel chains [43], human development [37], and water quality evaluation [38].

## 4. Conclusions

This research offers a solution to a widely recognized and controversial problem in the composite indicator literature: sub-indicators weighting. The research proposes a novel hybrid weighting method that maximizes the discriminating power of the composite indicator with objectively defined weights. It uses experts’ uncertainty about the conceptual importance of sub-indicators in the multidimensional phenomenon as maximum and minimum weights (constraints) in the optimization function.

The subjective–objective weighting scheme of the SAW-Max-Entropy method avoids assigning weights incompatible with the theoretical framework of the multidimensional phenomenon, as observed in purely objective weighting schemes. Its hybrid weighting scheme is also valuable for reducing the influence of assessment errors and judgment biases on composite indicator scores, which occur in purely subjective weighting schemes.

The results of this research represent a significant advance in comparison to studies limited to comparing composite indicators constructed by the Entropy Index with other weighting schemes [70,71,72], merging weights obtained by the Entropy Index with weights defined by experts [73], or increasing the discriminating power of the composite indicator after its construction [74].

The method has high application potential, considering the increasing use of composite indicators across diverse knowledge areas. The SAW-Max-Entropy method has broad appeal among researchers in the composite indicators field, as it effectively addresses the challenge of constructing composite indicators with greater discriminating power than other methods.

At least two lines of future investigations can be explored to enrich and improve the SAW-Max-Entropy method. Simulations can help measure the average gain in informational power of composite indicators constructed by the Max-Entropy weighting scheme compared to other weighting schemes. Developing software that automates the SAW-Max-Entropy method algorithm can be very useful in accelerating the dissemination of the method in academia, companies, and governments.

## Figures and Tables

**Table 1:** Performance of composite indicators constructed using a weighting scheme.

Weighting Scheme	Informational Diversity	Correlation with GDP	Rank Uncertainty	Consensus Degree
Entropy Index	0.17	−0.58	2.37	0.57
Expert Opinion	0.21	−0.74	2.37	1.00
Max-Entropy	0.24	−0.69	2.37	0.86
Equal Weights	0.19	−0.61	1.97	0.50

Note: The composite indicator’s informational diversity measure was calculated using the Entropy Index.

**Table 2:** Sub-indicator weights.

	Entropy Index	Expert Opinion	Max-Entropy	Equal Weights	Max. Weight	Min. Weight
Cp1.	0.06	0.13	0.02	0.06	0.29	0.02
Ec1.	0.05	0.03	0.02	0.06	0.07	0.02
Ec2.	0.04	0.07	0.11	0.06	0.29	0.02
Ec3.	0.07	0.04	0.02	0.06	0.15	0.02
Ec4.	0.11	0.05	0.02	0.06	0.15	0.02
Ge1.	0.11	0.03	0.02	0.06	0.07	0.02
Pt1.	0.05	0.12	0.14	0.06	0.29	0.05
Pt2.	0.02	0.07	0.14	0.06	0.15	0.02
Pt3.	0.03	0.16	0.23	0.06	0.29	0.03
Ri1.	0.02	0.03	0.02	0.06	0.10	0.02
Rp1.	0.05	0.03	0.02	0.06	0.07	0.02
St1.	0.07	0.06	0.05	0.06	0.15	0.02
St2.	0.07	0.07	0.12	0.06	0.29	0.02
Tb1.	0.05	0.03	0.02	0.06	0.04	0.02
Tb2.	0.07	0.03	0.02	0.06	0.04	0.02
Tb3.	0.07	0.03	0.02	0.06	0.05	0.02
Tb4.	0.06	0.03	0.02	0.06	0.05	0.02

## Data Availability

The data from the case study carried out in the research are available at https://data.mendeley.com/drafts/kwss6jyfxk, accessed on 23 January 2024.

## Appendix A

**Table A1:** Scores of the composite indicators constructed by different weighting schemes.

Country	Entropy	Experts	Max-Entropy	Equal-Weigths	GDP/Capita
Australia	0.05	0.03	0.16	0.09	52.85
Brazil	1.00	0.76	1.00	1.00	6.84
Canada	0.26	0.02	0.01	0.17	43.31
China	0.22	0.30	0.33	0.31	10.53
France	0.01	0.00	0.00	0.00	40.16
Germany	0.11	0.15	0.24	0.10	46.22
India	0.64	0.77	0.81	0.74	1.94
Indonesia	0.35	0.38	0.30	0.47	3.92
Iran	0.31	0.47	0.38	0.42	11.15
Italy	0.06	0.15	0.20	0.06	31.71
Japan	0.11	0.05	0.27	0.15	40.05
Korea. Rep.	0.19	0.20	0.19	0.17	31.64
Mexico	0.59	1.00	0.95	0.82	8.51
Netherlands	0.00	0.06	0.12	0.01	52.46
Russia	0.13	0.13	0.20	0.11	10.15
Spain	0.01	0.07	0.13	0.02	27.04
Switzerland	0.29	0.16	0.26	0.24	87.35
Turkey	0.61	0.53	0.37	0.58	8.61
United Kingdom	0.03	0.02	0.03	0.04	41.13
United States	0.22	0.04	0.06	0.20	63.08

Note: GDP/per capita in U.S. dollars.

**Table A2:** Ranking of countries in the composite indicators constructed by different weighting schemes.

Country	Entropy	Experts	Max-Entropy	Equal-Weigths	GDP/Capita
Australia	16	17	14	15	3
Brazil	1	3	1	1	18
Canada	8	18	19	11	6
China	10	7	6	7	14
France	18	20	20	20	8
Germany	14	11	10	14	5
India	2	2	3	3	20
Indonesia	5	6	7	5	19
Iran	6	5	4	6	13
Italy	15	10	11	16	10
Japan	13	15	8	12	9
Korea. Rep.	11	8	13	10	11
Mexico	4	1	2	2	17
Netherlands	20	14	16	19	4
Russia	12	12	12	13	15
Spain	19	13	15	18	12
Switzerland	7	9	9	8	1
Turkey	3	4	5	4	16
United Kingdom	17	19	18	17	7
United States	9	16	17	9	2

Note: GDP/per capita in U.S. dollars.
